# The efficacy and safety of hachimijiogan for mild Alzheimer disease in an exploratory, open standard treatment controlled, randomized allocation, multicenter trial: A study protocol

**DOI:** 10.1097/MD.0000000000022370

**Published:** 2020-09-18

**Authors:** Mosaburo Kainuma, Kouta Funakoshi, Shinji Ouma, Ken-ichiro Yamashita, Tomoyuki Ohara, Aoi Yoshiiwa, Masayuki Murata, Yoshio Tsuboi

**Affiliations:** a Community Medicine Education Unit, Graduate School of Medical Sciences, Kyushu University, Fukuoka, Japan; b Department of Clinical Research Promotion, Kyushu University Hospital, Fukuoka, Japan; c Department of Neurology, School of Medicine, Fukuoka University, Fukuoka, Japan; d Department of Clinical Neurophysiology, Faculty of Medicine, Graduate School of Medical Sciences, Kyushu University, Fukuoka, Japan; e Department of Neuropsychiatry, Graduate School of Medical Sciences, Kyushu University, Fukuoka, Japan; f Department of General Medicine, Oita University, Faculty of Medicine, Oita, Japan; g Department of General Internal Medicine, Kyushu University Hospital, Japan.

**Keywords:** ADAS-Jcog, Alzheimer disease, cognitive dysfunction, hachimijiogan, Kampo medicine

## Abstract

**Background:**

Dementia among the Japanese aged 65 years or over population is estimated to approach about 700 million cases by 2025, and a corresponding rapid increase in Alzheimer disease (AD) can also be expected. The ballooning number of dementia patients, including AD, is creating major medical and social challenges. At present, only 3 drugs are recognized for the treatment of mild AD, and these are only used to alleviate symptoms. Although new therapies are needed to treat mild AD, insufficient development of disease-modifying drugs is being done.

**Methods/Design:**

The aim of this exploratory, open standard, treatment-controlled, randomized allocation, multicenter trial is to determine the efficacy of the traditional Japanese Kampo medicine hachimijiogan (HJG) on the cognitive dysfunction of mild AD.

Eighty-six patients with AD diagnosed according to the Diagnostic and Statistical Manual of Mental Disorders (DSM)-5 and as mild AD according to the Mini Mental State Examination (MMSE ≥21) will be included. All will already have been taking the same dose of Donepezil, Galantamine, or Rivastigmine for more than 3 months. The patients will be randomly assigned to receive additional treatment with HJG or to continue mild AD treatment without additional HJG. The primary endpoint is the change from baseline of the Alzheimer's Disease Assessment Scale-cognitive component- Japanese version (ADAS-Jcog). ADAS-Jcog is a useful index for detecting change over time that investigates memory and visuospatial cognition injury from the early stage. The secondary endpoints are the changes from baseline of the Instrumental Activity of Daily Life, Apathy scale, and Nueropsychiatric Inventory scores. In this protocol, we will examine the Geriatric depression scale and do Metabolome analysis as exploratory endpoints. The recruitment period will be from August 2019 to July 2021.

**Discussion:**

This is the first trial of Kampo medicine designed to examine the efficacy of HJG for the cognitive dysfunction of patients with mild AD.

**Trial registration:**

This trial was registered on the Japan Registry of Clinical trials on 2 August 2, 2019 (jRCTs 071190018).

## 1 Introduction

Japan has become a super-aged society in which the elderly have become 1 of 4 people. The ballooning number of dementia patients is creating major medical and social challenges. It has been estimated that dementia will affect about 700 million people in the 65 or over population in 2025, and a corresponding rapid increase of Alzheimer disease (AD) can also be expected. Because the elderly population in Japan will continue to increase, comprehensive measures to combat dementia are an urgent national issue. AD is a disease that continues over the long-term, like lifestyle-related diseases. It develops through a preclinical phase without clinical symptoms but with mild cognitive impairment in the precursor stage. Considering the various problems that AD causes the affected individuals and their families and the increasing associated personal and social costs as dementia progresses, it has been proposed that early detection, early intervention, and primary prevention to prevent the onset of disease are necessary.^[1]^

Alois Alzheimer first reported a case of AD to the German Psychiatry Association in 1906. Even though over 100 years have passed, the etiology of AD has not been elucidated and no fundamental treatment regimen has been universally adopted, such as effective disease-modifying drugs. The current treatment method is based on the alleviation of symptoms, for which 3 acetylcholinesterase (AChE) inhibitors and 1 N-Methyl-D-Aspartate (NMDA) receptor antagonist have been approved. AD has been shown to worsen about 6 months after the introduction of AChE inhibitors.^[2,3]^ NMDA receptor antagonist is not indicated for mild AD as seen in the early stage, and no other pharmacological interventions at present are available for use until the disease state has progressed to a moderate or higher level in cases of redeterioration after AChE inhibitor introduction.

Of the AChE inhibitors, only donepezil has been confirmed to be effective for indices including activities of daily living, and there is insufficient evidence for the other 2 drugs.^[4,5]^ In the case of donepezil, a survey of therapeutic drugs prescribed to a large number of patients showed that the continuation rates after 6 and 12 months were extremely low, at 64.5% and 40.0%, respectively, because of uncertain effects.^[6]^ The development of new symptom-ameliorating drugs for mild AD is important.

Kampo medicines are produced uniquely in Japan and have been approved by the Ministry of Health, Labor and Welfare of Japan for the treatment of numerous diseases.

Hachimijiogan (HJG) is a traditional Japanese Kampo medicine that is prescribed for the treatment of “kidney deficiency,” a concept of Kampo medicine. It comprises 8 herbal components, RR, Cornus fruit, DR, AR, PS, MB, CB, and Aconite root, and it is effective for various age-related diseases, possibly including cognitive dysfunction, which is considered to be one of the symptoms of “kidney deficiency.” There is only 1 report on HJG. It found that it was effective for moderate to severe dementia (AD and cerebrovascular disease), but unfortunately, the number of patients was too small to be conclusive.^[7]^ We recently showed that memory impairment in CI + Aβ rats was ameliorated by HJG administration after ischemia treatment.^[8]^ Further, we have shown that HJG in vitro exerts a neurotrophic effect via cAMP response element binding protein (CREB) activation, and that HJG ameliorates cognitive dysfunction in dementia model rats via CREB activation.^[9]^

## 2 Methods

### 2.1 Objectives

The aim of this study is to verify the effect and safety of HJG on the cognitive dysfunction of mild AD patients.

### 2.2 Study design

**Figure 1:**
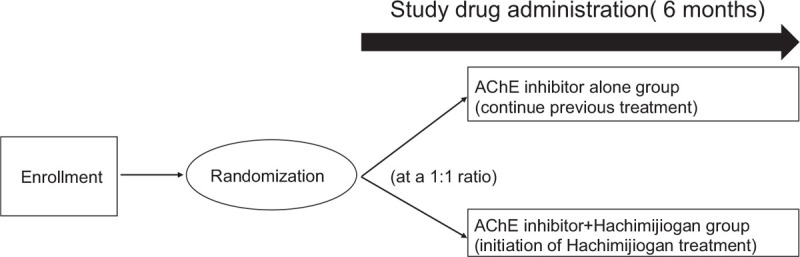
Study design. AChE = acetylcholinesterase.

Recommendations for Intervention Trials (SPIRIT) guidelines were followed for this protocol.^[10]^ The present study is an open-label, randomized, multicenter trial aiming to evaluate the efficacy and safety of the addition of HJG to any conventional AChE inhibitor (Dopepezil, Galantamine, Rivastigmine) as compared with continued use of AChE inhibitor alone in patients who have been diagnosed with AD according to the Diagnostic and Statistical Manual of Mental Disorders (DSM)-5 and with mild AD according to the Mini Mental State Examination (MMSE ≥21).^[11]^ The study will be conducted by the Department of Neuropsychiatry and Neurology of Kyushu University Hospital, the Department of Neurology of Fukuoka University Hospital, and the Department of General Medicine of Oita University Hospital. In total, 86 patients aged 50 to 84 years with mild AD will be randomly assigned to receive additional treatment with HJG or to continue treatment without additional HJG. The study design is summarized in Figure 1. The subjects will be randomly divided into 2 groups using stratified block randomization. For 6 months, 1 group will take HJG 7.5 g/day in powder form in addition to the previous standard treatment and the other group will continue to take only the previous standard treatment.

### 2.3 Study drug

The study drug [TSUMURA hachimijiogan (TJ-7) Extract Granules for Ethical Use] will be administered at a dose of 2.5 g three times per day (total daily dose of 7.5 g). TJ-7 is manufactured by Tsumura & Co. (Tokyo, Japan).

### 2.4 Inclusion criteria

Patients must meet all of the following requirements to be considered for entry into the study: age: ≥50 to <85 years, mild AD (MMSE≥21), taking the same dose of Donepezil, Galantamine, or Rivastigmine for more than 3 months, not taking Memantine, and written informed consent

### 2.5 Exclusion criteria

To avoid influencing the efficacy assessments, we will exclude patients who have been taking Kampo Medicine other than HJG for more than 3 months. Also excluded will be patients who have a change in drug dosage that could affect the progression of cognitive function during the 3 months. The other major exclusion criteria are as follows: Kidney disfunction (estimated glomerular filtration rate <30 mL/min/1.73 m^2^), aspartate aminotransferase or alanine aminotransferase >100 IU/L, Complication with gastric ulcer, bronchial asthma, or epilepsy, and Judged by doctors not to be suited for study, such as having serious complications.

### 2.6 Discontinuation criteria

The discontinuation criteria are as follows: When a subject declines to participate or withdraws consent, When the principal investigator or a research investigator determines that discontinuing the study is appropriate, When it is judged that an unfavorable event, such as a disease, has developed that makes it difficult to continue, When the administration of HJG is stopped, When the entire research plan is canceled, and If concomitant contraindications are used during the study period.

### 2.7 Outcome measurement

**Figure 2:**
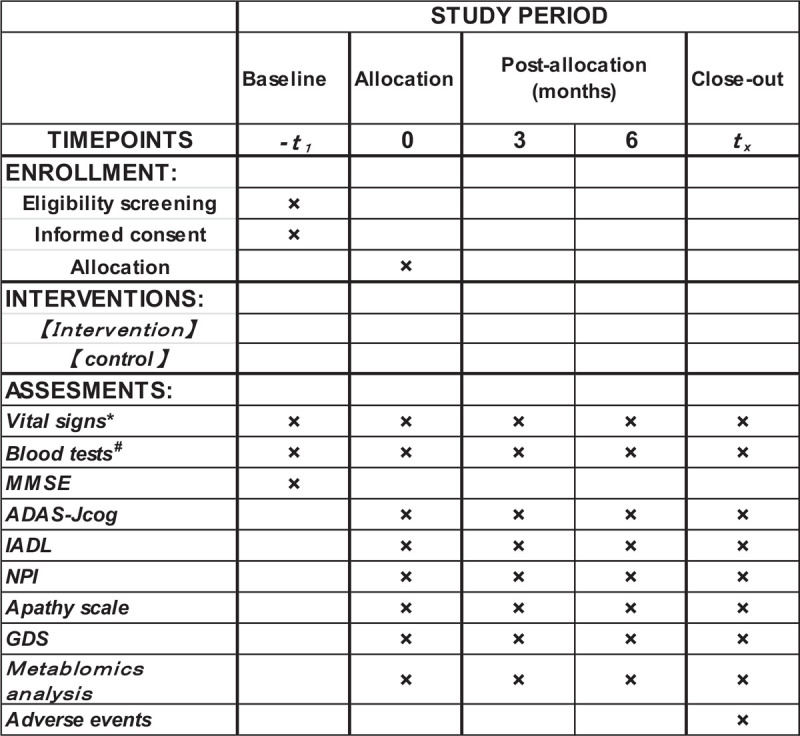
Schedule of enrollment, intervention, and assessment. ADAS-Jcog = Alzheimer's Disease Assessment Scale-cognitive component-Japanese version, GDS = Geriatric depression scale, IADL = Instrumental Activity of Daily Life, MMSE = Mini Mental State Examination, NPI = Neuropsychiatric Inventory. ^∗^Vital sign: Blood pressure and pulse rate. ^†^Blood tests: hematological examination and blood chemistry test.

Study visits will take place at baseline and after 3 and 6 months. The assessment schedule is represented in Figure 2. The primary endpoint is the change from baseline to month 6 of the Alzheimer's Disease Assessment Scale Cognitive Component- Japanese Version (ADAS-Jcog). ADAS-Jcog is a battery that assesses memory and visuospatial cognition injury from the early stage that is useful as an index for detecting change over time.^[12]^ Furthermore, because ADAS-cog is commonly used for assessing the efficacy of treatment, we selected the ADAS-Jcog as the primary end point. Guidelines on medicinal products for the treatment of AD and other dementias of the European Medicines Agency recommend the evaluation of cognition 169 days after treatment.^[13]^ and that most other drugs for AD also be evaluated at 169 days.

The secondary endpoints are score change from baseline of the Instrumental Activity of Daily Life (IADL), the Apathy scale, and the Neuropsychiatric Inventory (NPI). HJG is used for “Kidney deficiency” associated with various diseases related to age. We hypothesize that patients taking HJG will have improved ADL, and IADL will be used as the ADL score. Low motivation is sometimes associated with dementia, and cerebrovascular disease sequelae patients who took HJG for 4 weeks were reported to show significant improvement on the Apathy scale,^[14]^ which we will use for the evaluation of low motivation. NPI score is used for Behavioral and Psychological Symptoms of Dementia. Ohsawa et al^[15]^ reported that ninjinyoeito significantly improved the NPI score, and we added the change in NPI score as a secondary end point.

The exploratory endpoints are follows: The Geriatric depression scale (GDS) score, used for screening the depression of older people and metabolome analysis, used for biomarkers for the effect of HJG.

### 2.8 Randomization

Patients who meet the inclusion and exclusion criteria and have given written informed consent will be randomized after the screening consultation by use of Randomization Module of Research Electronical Data Capture (RED Cap). RED Cap is a secure web interface that is housed on secure severs and that has data checks during data entry and uploading to ensure data quality.^[16]^ The allocation sequence is a computer-generated list of random numbers transferred to RED Cap by a collaborator with no clinical involvement in the trial. Participants will be randomly assigned to either addition of HJG to any conventional AChE inhibitor or AChE inhibitor alone in a 1:1 ratio with stratification based on age (≤65 years and >65 years) and sex.

Participants and physicians are aware of the allocation arm after randomization. Data assessors are not blinded, but outcome assessors are blinded.

### 2.9 Data collection

Data will be recorded and stored via RED Cap as the study is in progress, and the data will be exported from the database into statistical software for analysis. In addition, RED Cap has an audit trail that records every time a participant or staff member makes changes to any data entered on the website.

### 2.10 Sample size consideration

We estimated the 6-month mean change from baseline of ADAS-cog in the AchE inhibitor arm as -1.78 ± 5.03, based on a prior clinical trial.^[17]^ The study as planned is exploratory, and there is insufficient evidence of the effect of HJG on mild AD to accurately calculate the sample size. To compensate, we initially planned to use the minimally clinically important difference (MCID) of ADAS-cog for calculation; however, because there were no prior studies about the MCID for ADAS-cog, we substituted the minimally important difference of ADAS-cog for each patient with mild AD. Schrag and Schott^[18]^ reported a score of 3 as the minimal clinically relevant change. By this method, the smallest number of subjects required for detecting the MCID between the treatment groups at a power of 80% and a 2-tailed significance level of 0.05 is calculated to be 72 (36 per group). Allowing for 15% drop-out, we will attempt to recruit 86 patients.

### 2.11 Adverse events

All adverse events (AEs) that occur between the administration of HJG and the end of month 6 will be recorded. A serious AE (SAE) is defined as any adverse reaction resulting in any of the following outcomes: a life-threatening condition or death or a condition that requires inpatient hospitalization or prolongation of an existing hospitalization, threatening to cause disability. Any SAEs will be documented in the medical records and be reported to the CRB by the responsible investigator, in accordance with Japanese regulations. If necessary, the investigators will administer treatment for an AE.

### 2.12 Statistical considerations

Efficacy analysis will be carried out on both the full analysis (FAS) and per-protocol sets (PPS). FAS is defined as follows: a subset of the enrolled subjects with the following excluded: significant noncompliance with the Clinical Research Law, untreated patients, and unobserved cases (completely missing the primary endpoint). PPS is defined as a subset of the subjects in the full analysis set with the following excluded: subjects who do not meet the specified selection criteria or who violate the exclusion criteria, severe deviation from the protocol, and subjects who are not compliant to treatment (compliance ratio <75%).

For the baseline characterization of the population, FAS will be tabulated for each treatment group. Descriptive statistics will be shown for continuous data, and the frequency and ratio (%) will be shown for categorical data.

#### 2.12.1 Analysis of the primary and secondary endpoints

Analysis of covariance will be used for the PPS, with the baseline value as a covariate. The same analysis will be done for FAS as a sensitivity analysis, and the robustness of the results will be confirmed. The mean value and standard deviation at each time point will be calculated for each treatment group. The analysis of secondary endpoints will be done with the same analysis as for the primary endpoint of the PPS.

#### 2.12.2 Safety analysis

The group consisting of subjects who receive the test drug at least once will be referred to as the Safety Analysis Set (SAS). For SAS, the number and incidence of hepatic dysfunction, interstitial pneumonia, and gastrointestinal disorders will be calculated for each treatment group.

### 2.13 Monitoring

Monitoring will be done periodically to ensure that this research is carried out safely and in accordance with the implementation plan, as well as to determine if data are accurately recorded and stored.

### 2.14 Ethics approval and consent to participate

This trial is being conducted at Kyushu University, Fukuoka University, and Oita University, Japan. All participants will be required to sign a written Consent Form. Before consent is obtained, the investigators will provide an oral explanation of the study to each participant. Participation is voluntary, and the patient's privacy will be protected. The participants will be informed of their right to withdraw from this study at any time and that this will not affect their clinical care. If this study poses a related serious health risk to a participant, compensation will be provided. The study has been approved by the Kyushu University Hospital Clinical Research Board Review (CRB) (CRB approval number:KD 2019001) and will be conducted in accordance with the World Medical Association Declaration of Helsinki of1996. The study results will be published in a peer-reviewed journal and be presented at national and international conferences.

## 3 Discussion

The current paper describes the protocol of an exploratory, open standard, treatment-controlled, randomized allocation, multicenter trial to investigate the efficacy of HJG on the cognitive dysfunction of patients with mild AD. Previous HJG study has been done with moderate to severe dementia with vascular and AD. This will be the first study to elucidate the effect of HJG on mild AD.

Kampo medicines, such as HJG, are commonly used for a wide variety of diseases in Japan. The drugs are relatively inexpensive and usually used alone or in combination with mainstream medicines. In clinical practice, most Japanese doctors prescribe Kampo Medicine in cases for which mainstream medicine is not so effective. The protocol for this study was designed to show the effect of adding the Kampo HJG to a mainstream AChE inhibitor.

Although HJG is mainly used for elderly patients, our experience is that HJG is also effective for young to middle-aged patients. We hypothesize that HJG will be effective for early-onset AD, so we set the target age at 50 years and older. In addition to HJG, it has been reported that the Kampo medicines kihito and ninjinyoeito improve the cognitive function of AD patients.^[15,19]^ However, all of the previous studies used MMSE to estimate cognitive function. Advanced cognitive function tests such as ADAS-cog will be necessary to make a more accurate evaluation, thus we set the change of ADAS-Jcog as the primary endpoint.

Because HJG consists of 8 constituent herbal medicines, it has a multifaceted effect. We speculate that the use of HJG would reduce the burden of caregivers, so the secondary endpoint will be estimation of efficacy by the NPI score.

This trial will help inform the development of a future large-scale RCT of this combined HJG and AChE inhibitor treatment for mild AD.

### 3.1 Trial status

The protocol version will be ver1.5 from June 16, 2020. The trial is currently in the participant recruitment stage, which began on August 2, 2019. Recruitment is expected to be completed on July 31, 2021.

## Acknowledgments

The authors would like to acknowledge Mieko Inada for her insightful suggestions and comments on our study protocol, and Kenji Sakanashi, Takao Yoshida, Shinichi Kijima, and Saki Hirata for their assistance to make the RED Cap.

## Author contributions

**Conceptualization:** Mosaburo Kainuma, Kouta Funakoshi, Shinji Ouma, Ken-ichiro Yamashita, Tomoyuki Ohara, Aoi Yoshiiwa, Masayuki Murata, Yoshio Tsuboi.

**Funding acquisition:** Mosaburo Kainuma.

**Investigation:** Shinji Ouma, Ken-ichiro Yamashita, Tomoyuki Ohara, Aoi Yoshiiwa, Yoshio Tsuboi.

**Methodology:** Kouta Funakoshi, Masayuki Murata.

**Project administration:** Mosaburo Kainuma.

**Writing – original draft:** Mosaburo Kainuma.

**Writing – review & editing:** Kouta Funakoshi, Shinji Ouma, Ken-ichiro Yamashita, Tomoyuki Ohara, Aoi Yoshiiwa, Masayuki Murata, Yoshio Tsuboi.

## Abbreviations

Abbreviations: AChE = acetylcholinesterase, AD = Alzheimer disease, ADAS-Jcog = Alzheimer's Disease Assessment Scale-cognitive component-Japanese version, AEs = All adverse events, AR = Alismatis rhizome, CB = Cinnamon bark, CREB = cAMP response element binding protein, DR = Dioscorea rhizome, FAS = full analysis set, GDS = Geriatric depression scale, HJG = Hachimijiogan, IADL = Instrumental Activity of Daily Life, jRCTS = Japan Registry of Clinical trials, MB = Moutan bark, MCID = minimally-clinically important difference, MMSE = Mini Mental State Examination, NPI = Neuropsychiatric Inventory, PPS = per-protocol set, PS = Poria Sclerotium, RED Cap = Research electronical data capture, RR = Rehmannia root, SAS = Safety analysis set.
